# Impact of COVID-19 pandemic on ocular disease: KNHANES 2015–2021

**DOI:** 10.1038/s41598-024-70767-y

**Published:** 2024-09-05

**Authors:** Hyeon Jung Kim, Yun Kyoung Ryu, Young Joo Shin

**Affiliations:** 1 Department of Ophthalmology, Kangnam Sacred Heart Hospital, Hallym University College of Medicine, Hallym University Medical Center, 1 Shingil-Ro, Youngdeungpo-Gu, Seoul, 07441 Republic of Korea; 2 Hallym BioEyeTech Research Center, College of Medicine, Hallym University, Seoul, Republic of Korea

**Keywords:** COVID-19 pandemic, Ocular diseases, Risk factor, Eye diseases, Risk factors

## Abstract

The aim of this study was to evaluate the impact of COVID-19 on ocular diseases and changes in risk factors before and after the COVID-19 pandemic. This study was conducted using data from the Korea National Health and Nutrition Examination Survey (KNHANES) 2015–2021, a national cross-sectional health examination and survey. Associations between ocular diseases and risk factors were determined using the chi-squared test and logistic regression analysis. Bivariable adjusted logistic regression analysis was performed to examine the odds ratio (OR) and 95% confidence interval (CI) to evaluate of the impact of COVID-19 on ocular diseases. Individuals were divided into two age groups (< 60 and ≥ 60 years). A total of 50,158 people were diagnosed, of whom 7270 were diagnosed with cataract, 921 with glaucoma, and 439 with age-related macular degeneration (AMD). Risk factors for cataract were COVID-19 pandemic (OR 1.161), hypertension (OR 1.608), diabetes (OR 1.573), dyslipidemia (OR 1.167), stroke (OR 1.272), and depression (OR 1.567). Risk factors for AMD were COVID-19 pandemic (OR 1.600), dyslipidemia (OR 1.610), and depression (OR 1.466). Risk factors for glaucoma were hypertension (OR 1.234), dyslipidemia (OR 1.529), diabetes (OR 1.323), and depression (OR 1.830). The COVID-19 pandemic was a risk factor for cataracts and AMD, but not for glaucoma. Cataracts and AMD may be more influenced by the acquired health conditions or the environment.

## Introduction

COVID-19 is an infectious disease caused by a newly discovered coronavirus^1^. It is a novel strain of the virus that had not been previously identified in humans and has since spread throughout the world^2^. Symptoms of the disease include cough, fever, shortness of breath, and fatigue^2^. COVID-19 pandemic has had a major impact on daily activities around the world to help slow the spread of the virus and protect public health. Many countries have implemented social distancing measures, such as stay-at-home orders, travel restrictions, and business closures^3^. Schools and universities have been closed, and many people are now working from home. Changes in lifestyle patterns due to COVID-19 pandemic have affected nutritional status and physical activities^4^.

The most common ocular diseases were cataract, glaucoma, and age-related macular degeneration (AMD)^5^. The prevalence of cataract was the highest of all the ocular diseases studied, followed by glaucoma and AMD, with a marked increase in the prevalence of both glaucoma and AMD over time^5^. Although they are associated with a number of risk factors, it is not clear whether the COVID-19 pandemic has an impact on ocular disease such as cataract, glaucoma and AMD. However, the prevalence of risk factors such as obesity, dyslipidemia, hypertension, diabetes, smoking, reduction in physical activity and stroke, depression have increased with COVID-19 pandemic^6,7^. As COVID-19 has caused changes in the immune system and lifestyle habits^8^, it is possible that the prevalence of eye diseases has changed. Therefore, it was necessary to find out the changes in the prevalence of eye diseases and the differences in risk factors due to COVID-19. Investigating the impact of the COVID-19 pandemic on the prevalence of eye disease and the associated risk factors is essential to understanding the multiple ways in which this disease affects human health. Research into the impact of COVID-19 on ocular health may help to improve prevention and treatment methods, and to gain a better understanding of the long-term effects of this disease. The aim of this study was to evaluate the effect of COVID-19 on ocular disease in a Korean population.

## Methods

The Korean National Health and Nutrition Examination Survey (KNHANES) was approved by the Institutional Review Board (IRB) of the Korean Centers for Disease Control and Prevention (2013-12EXP-03-5C, 2013-12EXP-03-5C, 2018-01-03-C-A, 2018-01-03-2C-A, 2018-01-03-5C-A, and 2018-01-03-4C-A). This study adhered to the tenets of the Declaration of Helsinki and was approved from IRB approval by the institutional review board of Hallym University Kangnam Sacred Heart Hospital (2024-04-011).

**Fig. 1:**
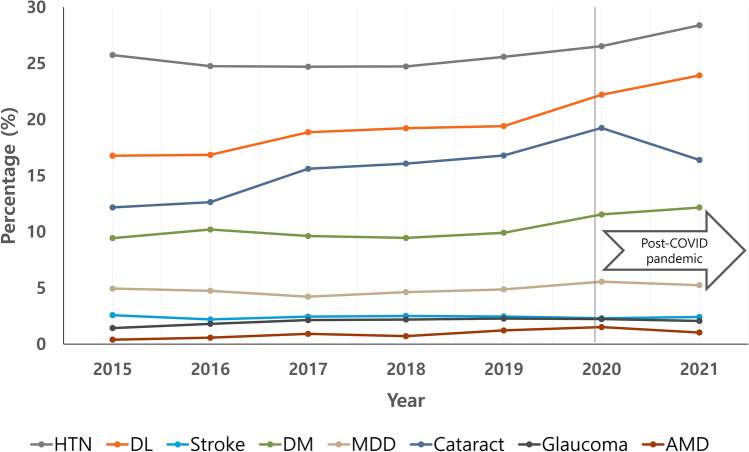
Disease prevalence by year.

For this cross-sectional, population-based study, we used data from the KNHANES 2015–2021, a series of cross-sectional surveys of nationally representative samples of the Korean civilian population, conducted annually to assess the health and nutrition status of the South Korean population. To obtain representative samples, KNHANES uses a stratified, multistage, cluster probability sampling design by geographical area, age, and sex. For the health interview survey, a trained interviewer asked questions directly to individuals aged ≥ 19 years old. The inclusion criteria for this study were: (1) adults over 19 years of age, (2) those who completed a questionnaire on independent risk factors. Our exclusion criteria were aged < 19 years. Subjects were asked whether they had been diagnosed with cataracts by an ophthalmologist, in the same way as they were asked whether they had been diagnosed with cataract, glaucoma or AMD. Risk factors such as diabetes, hypertension, dyslipidemia, major depression disorder (MDD), and aerobic physical activity were questioned. Incorrect or untested responses (untested) were excluded (Fig. 1). The population was divided according to age: young (< 60 years) or older (≥ 60 years). The pre-COVID-19 period was 2015–2019, and the post-COVID-19 period was set at 2020–2021.

Statistical analyses were performed using SPSS Version 27.0 (SPSS Inc., IBM Software, Portsmouth, UK), and two-tailed p-values less than 0.05 were considered statistically significant. To estimate the odds ratios (ORs) of cataract and potential factors, we performed binary logistic regression analyses using a generalized linear model for a complex survey design. The ORs measure the odds of an outcome occurring in the presence of various risk factors compared to the odds of the outcome occurring in their absence. ORs are presented with corresponding 95% confidence intervals (CIs). CIs provide a range of values that are believed to cover the true value of the ORs to a 95% level of confidence, thus giving an indication of the precision of our estimates.

### Institutional review board statement

This study was approved by the Institutional Review Board of Kangnam Sacred Heart Hospital (Approval number: 2024-04-009).

## Results

**Fig. 2:**
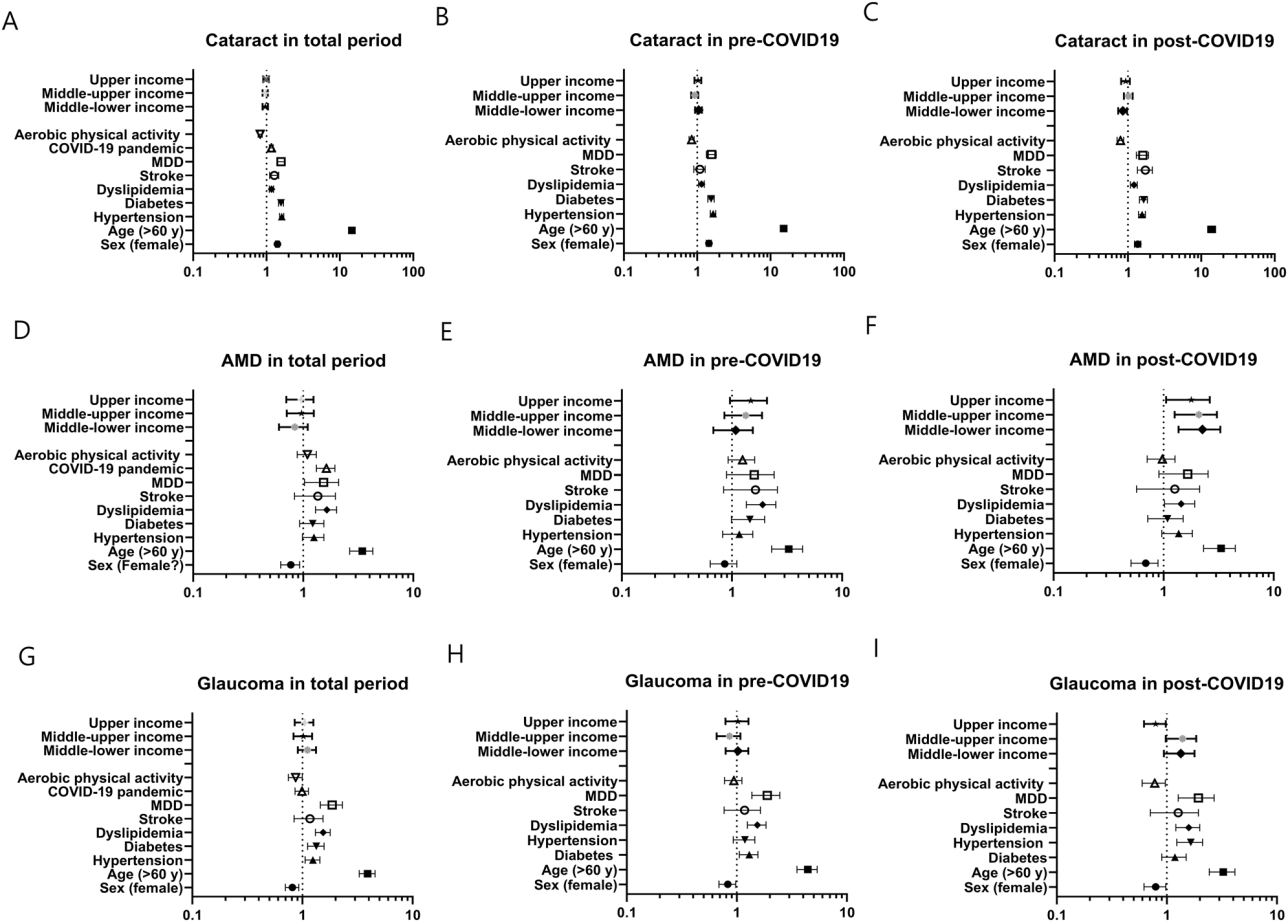
Forest plots showing the odds ratio with 95% confidence interval. (**A**–**C**) The association between cataracts and risk factors in total study periods, pre-COVID-19 and post-COVID-19. (**D**–**F**) The association between AMD and risk factors in total study periods, pre-COVID-19 and post-COVID-19. (**G**–**I**) The association between glaucoma and risk factors in total study periods, pre-COVID-19 and post-COVID-19.

The characteristics of the study population are shown in Table 1. Of the 61,567 people in KHANES 2015–2021, 50,158 people (22,284 men and 27,874 women) over 19 years of age were included in this study, excluding 11,409 under 19 years of age. Of these, 33,073 were pre-COVID-19 and 17,085 were post-COVID-19. Of these, 7270 were diagnosed with cataracts, 921 with glaucoma and 439 with AMD. Mean age was 51.88 ± 17.09 years. Disease prevalence by year is shown in Table 1 and Fig. 1. We found the prevalences of cataract and AMD increased in post-COVID-19 compared to pre-COVID-19 Risk factors for cataract were COVID-19 pandemic (OR 1.161; 95% CI 1.091–1.235), hypertension (OR 1.608; 95% CI 1.506–1.717), diabetes (OR 1.573; 95% CI 1.455–1.701), dyslipidemia (OR 1.167; 95% CI 1.091–1.249), stroke (OR 1.272; 95% CI 1.105–1.463), and depression (OR 1.567; 95% CI 1.391–1.765) (Table 2 and Fig. 2A). Aerobic physical activity was the protective factor (OR 0.820; 95% CI 0.769–0.874). Before the COVID-19 pandemic, risk factors for cataract were sex (OR 1.437; 95% CI 1.327–1.557), age (OR 14.971; 95% CI 13.518–16.580), hypertension (OR 1.641; 95% CI 1.509–1.785), diabetes (OR 1.542; 95% CI 1.394–1.706), dyslipidemia (OR 1.144; 95% CI 1.048–1.249), and MDD (OR 1.558; 95% CI 1.337–1.815), not stroke (OR 1.075; 95% CI 0.900–1.284) (Table 2 and Fig. 2B). Aerobic physical activity was a protective factor (OR 0.837; 95% CI 0.772–0.909). However, stroke and personal income was not significant factor. After the start of the COVID-19 pandemic, stroke (OR 1.700; 95% CI 1.346–2.147) was found to be an important risk factor in addition to existing risk factors and middle-lower income to be a protective factor (Table 2 and Fig. 2C).

**Table 1:** Demographic data.

	Total	Pre-COVID-19	Post-COVID-19	p-value
N	50,158	33,073	17,085	
Period	2015–2021	2015–2019	2020–2021	
Age (year)	51.88 ± 17.09	51.54 ± 16.90	52.53 ± 17.42	< 0.001*
Sex (M:F)	22,284:27,874	14,714:18,359	7570:9515	0.704
Cataract	7270 (16.0%)	4477 (15.0%)	2793 (18.1%)	< 0.001*
Glaucoma	921 (2.0%)	599 (2.0%)	322 (2.1%)	0.419
AMD	439 (1.0%)	236 (0.8%)	203 (1.3%)	< 0.001*

*AMD* age-related macular degeneration. *p <0.05

**Table 2:** The risk factors for cataract at pre- and post-COVID-19 pandemic.

General Characteristics	OR	95% CI		p-value
		Lower	Upper	
Total				
Sex (female)	1.404	1.319	1.494	< 0.001*
Age (> 60 years)	14.489	13.386	15.684	< 0.001*
Hypertension	1.608	1.506	1.717	< 0.001*
Diabetes	1.573	1.455	1.701	< 0.001*
Dyslipidemia	1.167	1.091	1.249	< 0.001*
Stroke	1.272	1.105	1.463	< 0.001*
MDD	1.567	1.391	1.765	< 0.001*
COVID-19 pandemic	1.161	1.091	1.235	< 0.001*
Aerobic physical activity	0.820	0.769	0.874	< 0.001*
Income				
Lower				0.732
Middle-lower	0.962	0.883	1.047	0.367
Middle-upper	0.958	0.880	1.043	0.323
Upper	0.983	0.903	1.071	0.699
Pre-COVID-19				
Sex (female)	1.437	1.327	1.557	< 0.001*
Age (> 60 years)	14.971	13.518	16.580	< 0.001*
Hypertension	1.641	1.509	1.785	< 0.001*
Diabetes	1.542	1.394	1.706	< 0.001*
Dyslipidemia	1.144	1.048	1.249	0.003*
Stroke	1.075	0.900	1.284	0.424
MDD	1.558	1.337	1.815	< 0.001*
Aerobic physical activity	0.837	0.772	0.909	< 0.001*
Income				
Lower				0.137
Middle-lower	1.042	0.936	1.161	0.452
Middle-upper	0.922	0.827	1.029	0.147
Upper	1.020	0.915	1.137	0.718
Post-COVID-19				
Sex (female)	1.357	1.228	1.498	< 0.001*
Age (> 60 years)	13.816	12.183	15.668	< 0.001*
Hypertension	1.552	1.397	1.725	< 0.001*
Diabetes	1.626	1.436	1.841	< 0.001*
Dyslipidemia	1.203	1.081	1.340	0.001*
Stroke	1.700	1.346	2.147	< 0.001*
MDD	1.577	1.303	1.909	< 0.001*
Aerobic physical activity	0.788	0.711	0.874	< 0.001*
Income				0.038*
Lower	1 (ref)			
Middle-lower	0.845	0.736	0.970	0.017*
Middle-upper	1.009	0.882	1.156	0.892
Upper	0.925	0.806	1.062	0.270

*MDD* Major depressive disorder, *p < 0.05.

Risk factors for AMD were the COVID-19 pandemic (OR 1.600; 95% CI 1.318–1.943), age (OR 3.367; 95% CI 2.643–4.289), dyslipidemia (OR 1.610; 95% CI 1.291–2.008), and depression (OR 1.466; 95% CI 1.026–2.097) and sex was a protective factor (OR 0.761; 95% CI 0.624–0.928) (Table 3 and Fig. 2D). Before the COVID-19 pandemic, risk factors for AMD included age (OR 3.160; 95% CI 2.287–4.367), and dyslipidemia (OR 1.837; 95% CI 1.350–2.500) (Table 3 and Fig. 2E). After the start of the COVID-19 pandemic, income was a significant risk factor whereas sex was a protective factor (OR 0.670; 95% CI 0.505–0.889) (Table 3 and Fig. 2F).

**Table 3:** Risk factors for AMD at pre- and post- COVID-19 pandemic.

General characteristics	OR	95% CI		p-value
		Lower	Upper	
Total				
Sex (Female)	0.761	0.624	0.928	0.007*
Age (> 60 years)	3.367	2.643	4.289	< 0.001*
Hypertension	1.231	0.983	1.541	0.070
Diabetes	1.195	0.928	1.540	0.168
Dyslipidemia	1.610	1.291	2.008	< 0.001*
Stroke	1.278	0.833	1.961	0.261
MDD	1.466	1.026	2.097	0.036*
COVID-19 pandemic	1.600	1.318	1.943	< 0.001*
Aerobic physical activity	1.076	0.880	1.315	0.477
Income				0.572
Lower	1 (ref)			
Middle-lower	0.814	0.603	1.098	0.178
Middle-upper	0.940	0.709	1.246	0.665
Upper	0.933	0.702	1.240	0.633
Pre-COVID-19				
Sex (female)	0.837	0.634	1.105	0.209
Age (> 60 years)	3.160	2.287	4.367	< 0.001*
Hypertension	1.128	0.822	1.546	0.456
Diabetes	1.398	0.986	1.982	0.060
Dyslipidemia	1.837	1.350	2.500	< 0.001*
Stroke	1.472	0.839	2.583	0.178
MDD	1.468	0.892	2.415	0.131
Aerobic physical activity	1.217	0.921	1.608	0.167
Income				
Lower				0.221
Middle-lower	1.023	0.678	1.545	0.913
Middle-upper	1.263	0.852	1.874	0.245
Upper	1.412	0.960	2.078	0.080
Post-COVID-19				
Sex (female)	0.670	0.505	0.889	0.005*
Age (> 60 years)	3.215	2.309	4.476	< 0.001*
Hypertension	1.325	0.963	1.822	0.084
Diabetes	1.038	0.717	1.504	0.842
Dyslipidemia	1.397	1.019	1.915	0.038*
Stroke	1.099	0.568	2.124	0.780
MDD	1.517	0.908	2.534	0.112
Aerobic physical activity	0.949	0.709	1.269	0.722
Income				0.006*
Lower	1 (ref)			
Middle-lower	2.115	1.366	3.275	0.001*
Middle-upper	1.960	1.260	3.050	0.003*
Upper	1.663	1.052	2.629	0.030*

*MDD* Major depressive disorder. *p <0.05

Risk factors for glaucoma were age (OR 3.864; 95% CI 3.272–4.563), hypertension (OR 1.234; 95% CI 1.056–1.441), diabetes (OR 1.323; 95% CI 1.113–1.572), dyslipidemia (OR 1.529; 95% CI 1.311–1.783), and MDD (OR 1.830; 95% CI 1.453–2.303), but not the COVID-19 pandemic. Protective factor for glaucoma was sex (OR 0.804; 95% CI 0.700–0.924) and aerobic physical activity (OR 0.861; 95% CI 0.745–0.996) (Table 4 and Fig. 2G). Before the COVID-19 pandemic, the risk factors were age (OR 4.343; 95% CI 3.512–5.372), hypertension (OR 1.280; 95% CI 1.052–1.558), dyslipidemia (OR 1.517; 95% CI 1.248–1.844), MDD (OR 1.838; 95% CI 1.372–2.461), and the protective factor was sex (OR 0.821; 95% CI 0.688–0.970) (Table 4 and Fig. 2H). Diabetes and aerobic physical activity were not significant factor. After the start of the COVID-19 pandemic, hypertension was not a risk factor and diabetes (OR 1.6129; 95% CI 1.231–2.111) was a risk factor. Aerobic physical activity was a protective factor (OR 0.760; 95% CI 0.597–0.968) (Table 4 and Fig. 2I).

**Table 4:** Risk factors for glaucoma at pre- and post-COVID-19 pandemic.

General characteristics	OR	95% CI		p-value
		Lower	Upper	
Total				
Sex (female)	0.804	0.700	0.924	0.002*
Age (> 60 years)	3.864	3.272	4.563	< 0.001*
Hypertension	1.234	1.056	1.441	0.008*
Diabetes	1.323	1.113	1.572	0.001*
Dyslipidemia	1.529	1.311	1.783	< 0.001*
Stroke	1.134	0.838	1.535	0.416
MDD	1.830	1.453	2.303	< 0.001*
COVID-19 pandemic	0.985	0.856	1.133	0.832
Aerobic physical activity	0.861	0.745	0.996	0.044*
Income				
Lower				0.763
Middle-lower	1.097	0.907	1.327	0.342
Middle-upper	1.006	0.828	1.222	0.952
Upper	1.032	0.849	1.255	0.750
Pre-COVID-19				
Sex (female)	0.821	0.688	0.979	0.028*
Age (> 60 years)	4.343	3.512	5.372	< 0.001*
Hypertension	1.280	1.052	1.558	0.014*
Diabetes	1.163	0.928	1.457	0.190
Dyslipidemia	1.517	1.248	1.844	< 0.001*
Stroke	1.120	0.766	1.638	0.559
MDD	1.838	1.372	2.461	< 0.001*
Aerobic physical activity	0.928	0.774	1.114	0.424
Income				
Lower				0.432
Middle-lower	1.004	0.791	1.273	0.975
Middle-upper	0.841	0.656	1.079	0.174
Upper	1.003	0.789	1.274	0.983
Post-COVID-19				
Sex (female)	0.779	0.621	0.977	0.030*
Age (> 60 years)	3.178	2.432	4.153	< 0.001*
Hypertension	1.158	0.897	1.494	0.260
Diabetes	1.612	1.231	2.111	0.001*
Dyslipidemia	1.549	1.206	1.990	0.001*
Stroke	1.169	0.708	1.932	0.541
MDD	1.846	1.269	2.685	0.001*
Aerobic physical activity	0.760	0.597	0.968	0.026*
Income				
Lower				0.213
Middle-lower	1.294	0.939	1.782	0.116
Middle-upper	1.344	0.978	1.847	0.068
Upper	0.779	0.621	0.977	0.621

*MDD* Major depressive disorder. *p < 0.05

## Discussion

The COVID-19 pandemic, caused by the novel coronavirus SARS-CoV-2, emerged in late 2019 and rapidly evolved into a global health crisis^9^. It has led to widespread disease and significant mortality, with a profound impact on public health, economies, and daily life worldwide^9^. In this study, we investigated the effect of the COVID-19 pandemic on the prevalence and risk factors for ocular disease. This study found that COVID-19 pandemic was an important risk factor for cataract and AMD, but not for glaucoma. There may be several possible reasons why the prevalence of cataract and AMD has increased since the COVID-19 outbreak. First, changes in diet and lifestyle due to the COVID-19 pandemic. Changes in lifestyle due to the pandemic may affect nutritional status and daily activities, which may also affect the risk factors for ocular diseases such as diabetes, dyslipidemia and hypertension due to restrictions on outdoor activities^10^. Second, with immune system changes, COVID-19 affects the patient's immune system. This may worsen existing eye diseases or increase the risk of developing new eye diseases. COVID-19 causes epigenetic changes and hyperactivation in monocytes and permanent changes in stem cell gene expression, causing the immune system to produce more white blood cells^11^. The third is the postponement or cancellation of regular eye examinations due to COVID-19. Many people have been unable to access regular health care during the pandemic, including the postponement or cancellation of eye examinations. This may have the delayed early detection and treatment of some eye diseases. Fourth, social isolation and stress: prolonged social isolation can have a negative impact on mental health, which can also affect physical health, especially eye health. Stress is known to be a risk factor for several eye diseases^12^. In addition, with many people spending more time at home due to social isolation, screen time on computers, smartphones and other devices has increased. This can lead to increased vision problems and eye fatigue^13^. Furthermore, oxidative stress, which is a main pathogenesis of cataract and AMD, is increased by COVID-19 infection and damages the lens and retinal pigment epithelium^14,15^. However, the COVID-19 pandemic is not a risk factor for glaucoma. Glaucoma is thought to be caused more by endogenous factors rather than exogenous factors. COVID-19 is caused by the SARS-CoV-2 virus, which primarily affects the respiratory system but can also impact other organs due to its inflammatory and vascular effects^16^. Glaucoma, on the other hand, is a group of eye conditions that primarily involve the degeneration of the optic nerve, often associated with increased intraocular pressure^17^. The etiology of glaucoma is complex, involving genetic, vascular, and mechanical components that are not directly influenced by viral infections like COVID-19^17^.

This study showed that risk factors have changed since the COVID-19 pandemic. Stroke was not a risk factor for cataract, but after the COVID-19 pandemic, it became an important risk factor. In AMD, dyslipidemia was a major risk factor rather than sex, but this was reversed after the COVID-19 pandemic. These changes in risk factors may be due to changes in the immune system caused by coronavirus infection. COVID-19 may trigger stronger inflammatory responses in men^18,19^. Glaucoma was not associated with diabetes and aerobic physical activity before the COVID-19 pandemic, but became associated after the COVID-19 pandemic. Lifestyle factors may become important in the management and reduction of glaucoma risk factors.

Surprisingly, household income had an effect on the change of risk factors. For cataracts, income was not a risk factor in pre-COVID-19, but upper-middle income was a protective factor for low income in post-COVID-19. For AMD, income was not a risk factor in pre-COVID-19, but an increase in income was a risk factor for low income in post-COVID-19. This is likely to have been influenced by the increase in home offices, long-time computer use, decline in outdoor physical activities, and social isolation among the upper-middle income class post-COVID-19 during the pandemic.

In conclusion, the COVID-19 pandemic was a risk factor for cataract and AMD, but not for glaucoma. Cataract and AMD may be more affected by the external factors than glaucoma. The indirect effects of the pandemic, such as stress, changes in immune system, changes in access to healthcare, and lifestyle changes, are likely to play a significant role in these observed trends.

## Data Availability

All the data utilized in this study are publicly available through the KNHANES website (http://knhanes.cdc.go.kr, accessed on 14 July 2022).
